# Accurate affinity models for SH2 domains from peptide binding assays and free‐energy regression

**DOI:** 10.1002/pro.70317

**Published:** 2025-10-14

**Authors:** Dejan Gagoski, H. Tomas Rube, Chaitanya Rastogi, Lucas A. N. Melo, Xiaoting Li, Rashmi Voleti, Neel H. Shah, Harmen J. Bussemaker

**Affiliations:** ^1^ Department of Biological Sciences Columbia University New York New York USA; ^2^ Department of Chemistry Columbia University New York New York USA; ^3^ Department of Applied Mathematics University of California‐Merced Merced California USA; ^4^ Department of Systems Biology Columbia University New York New York USA

**Keywords:** bacterial peptide display, binding affinity prediction, biophysically interpretable machine learning, next‐generation sequencing (NGS), SH2 domains

## Abstract

Short linear peptide motifs play important roles in phosphotyrosine‐dependent signaling networks. They can act both as substrates of kinases and phosphatases and as ligands of peptide binding domains. SH2 domains bind specifically to tyrosine‐phosphorylated proteins, with the affinity of the interaction depending strongly on the flanking sequence. In recent years, protein display technologies and next‐generation sequencing (NGS) have allowed researchers to profile SH2 domain binding across large libraries of candidate ligands. Here, we present a concerted experimental and computational strategy that updates such specificity profiling from classification to quantification. Multi‐round affinity selection on random phosphopeptide libraries yields NGS data suitable for training an additive model that accurately predicts binding free energy across the full theoretical ligand sequence space. For SH2 domains that have been profiled in this manner, the sequence‐to‐affinity model can be used to predict novel phosphosite targets or the impact of phosphosite variants on binding.

## 1 INTRODUCTION

Many protein–protein interactions in the cell occur between a peptide recognition domain (PRD) and a short linear peptide sequence, both of which are typically embedded within larger proteins (Tompa et al., 2014). These peptide sequences, often referred to as short linear motifs (SLiMs), can be bound by a variety of PRDs, with a binding energy that often depends strongly on the amino‐acid sequence of the peptide ligand. SLiMs play important roles in the formation of protein complexes and the regulation of signaling cascades. Paralogous PRDs from the same structural family can have distinct SLiM binding preferences indicative of functional specialization, despite their overall homology.

SLiMs may contain residues that can be post‐translationally modified, allowing their interactions to be regulated. Kinases that phosphorylate serine, threonine, or tyrosine residues in SLiMs have long been a subject of intense study (Yaffe et al., 2001), and their substrate sequence specificities have been extensively probed in high‐throughput (Johnson et al., 2023; Songyang et al., 1994; Yaron‐Barir et al., 2024). Src‐homology 2 (SH2) domains specifically bind to SLiMs containing a phosphorylated tyrosine (pY), and as such, they mediate regulated protein–protein interactions in response to tyrosine kinase activity.

Mutations within SLiMs can either weaken or strengthen the protein–protein interactions they mediate, allowing for rapid evolution of new signaling networks as well as the emergence of pathogenic molecular processes (Davey et al., 2015; Li et al., 2019; Meyer et al., 2018). Being able to predict the wiring and rewiring of SLiM‐based interaction networks in terms of the binding specificity of PRDs can enhance our fundamental understanding of cell signaling, shed light on the evolution of signaling pathways, reveal mechanisms of pathogenicity for uncharacterized mutations, and suggest novel therapeutic strategies.

Over the past two decades, many different experimental techniques for probing SLiM‐based interaction networks have been developed (Davey et al., 2023). Of particular interest are experimental methods that have been used to profile the ligand sequence specificity of SH2 domains, including affinity selection on pY‐oriented random phosphopeptide libraries coupled with classic peptide sequencing (Songyang et al., 1993), labeled SH2 domains incubated with pY‐oriented random peptide array libraries on cellulose filters (Huang et al., 2008; Rodriguez et al., 2004; Yaron‐Barir et al., 2024) or with arrays of defined phosphopeptides (Jones et al., 2006; Tinti et al., 2013), and high‐throughput affinity measurements in solution for large numbers of SH2‐peptide pairs (Hause Jr. et al., 2012; Leung et al., 2014).

SH2 specificity datasets have been exploited to build sequence‐based classifiers that can be used to rapidly scan protein sequences for the presence of SH2 binding sites, which is of great biological value. In their simplest form, these classifiers take the form of a position‐specific scoring matrix (PSSM); a score threshold is applied to distinguish between binders and non‐binders, and performance is scored in terms of false positive and false negative rates (Huang et al., 2008; Leung et al., 2014; Obenauer et al., 2003; Tinti et al., 2013). More sophisticated machine learning methods have also been used for this classification task (AlQuraishi et al., 2014; Kundu et al., 2013; Yin et al., 2024). However, none of these methods have been explicitly validated for their ability to *quantitatively* predict binding affinities (or, equivalently, binding free energies) in biophysically meaningful units. In this context, it is relevant to note that a previous systematic benchmarking of algorithms for inferring scoring matrices for protein‐DNA interaction revealed that discriminative approaches based on information theory performed less well than methods based on biophysical models (Weirauch et al., 2013).

The use of increasingly large and affordable DNA‐encoded protein/peptide display libraries has dramatically increased the scale and throughput of experiments to profile PRD‐SLiM interactions (Davey et al., 2017; Ivarsson et al., 2014; Teyra et al., 2020). Such phage display assays have also been coupled with next‐generation sequencing (NGS) (Benz et al., 2022; Ueki et al., 2019). Recently, several SH2 domains were analyzed using a combination of bacterial display of genetically‐encoded peptide libraries, enzymatic phosphorylation of the displayed peptides, affinity‐based selection, and NGS (Cantor et al., 2018; Li et al., 2023; van Vlimmeren et al., 2024). These studies employed a variety of library formats, including scanning mutagenesis libraries derived from individual peptides (<10^3^ sequences) (Cantor et al., 2018; van Vlimmeren et al., 2024), phosphoproteome‐derived peptide libraries (10^3^–10^4^ sequences) (Cantor et al., 2018; Li et al., 2023; van Vlimmeren et al., 2024), and degenerate libraries (10^6^–10^7^ sequences) (Li et al., 2023). With these methods and data in hand, there are new opportunities for building quantitative models of SH2 domain binding specificity.

**FIGURE 1:**
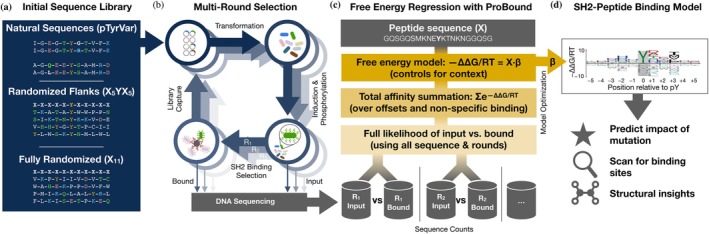
Overview of concerted experimental and computational strategy for generating SH2‐peptide binding free energy models. (a) Design of peptide‐display libraries. (b) Schematic showing how a randomized bacterial display library underwent repeated bead‐based affinity selection for SH2 binding. In each selection round, the library was sequenced before and after selection. (c) Overview of the regression framework used to learn energetic binding models from the sequencing data. For each possible binding site, the energy received independent additive contributions from the residues flanking the phosphorylated tyrosine, thus controlling for the binding‐site context wherein the residues reside. These energy contributions were estimated using maximum likelihood estimation, where the likelihood of the observed sequence counts was evaluated by first computing the total affinity for each observed sequence (controlling for multiple possible binding offsets and non‐specific binding) and then computing the binomial likelihood for each round, assuming linear section. (d) Sequence logo displaying the inferred energy contributions as letters whose height reflects the magnitude of the contributions, relative to the mean for each position.

Here, we report a coordinated experimental and computational strategy for analyzing sequence recognition by PRDs (Figure 1) that improves upon these pioneering studies. Our approach employs ProBound, a statistical learning method originally developed by our group to accurately model protein‐DNA interactions (Rube et al., 2022). As discussed below, ProBound is highly customizable, can accommodate extremely sparse coverage of sequences for highly complex libraries, can jointly analyze data from multi‐round selection experiments, and generates quantitative sequence‐to‐affinity models that cover the full theoretical sequence space and are not dependent on library format. Indeed, in a recent study, we showed that bacterial display of a random peptide library coupled with NGS, followed by ProBound analysis, allowed us to build an accurate sequence‐based predictor of enzymatic efficiency for the tyrosine kinase c‐Src (Rube et al., 2022). Focusing here on six representative SH2 domains, we combine peptide display, sequencing of highly degenerate random libraries, and ProBound to build models that accurately predict binding affinity for any ligand sequence in the theoretical space covered by the library. Our strategy should be readily adaptable to other peptide recognition domains.

## 2 RESULTS

### 2.1 An integrated experimental‐computational framework for generating sequence‐to‐affinity models for peptide recognition domains

The overall objective of this study was to leverage affinity selection of random peptide libraries to systematically profile the sequence specificity of SH2 domains. An attractive feature of such a method would be that many SH2 domains could be profiled using the same library. However, interpreting the resulting data is difficult due to a number of challenges stemming from the extreme sequence diversity of random libraries. For the input library, the effect of this diversity is that the sequencing counts for individual sequences are low and noisy, making it challenging to control for non‐uniformities in the input. While affinity selection focuses the library toward high‐affinity sequences, even perfect affinity selection (with enrichment proportional to binding affinity) leaves such high‐affinity sequences in the minority. This is because low‐affinity sequences are (i) far more numerous and (ii) subject to non‐specific binding and experimental carry‐over. While repeated selection focuses the library further, this introduces several new challenges: biases could be introduced when the library is captured and prepared for the next round, and low‐affinity sites are exponentially depleted over selection rounds, meaning excessive selection removes information about low‐affinity sequences. Beyond these challenges stemming from library diversity, even a perfect dataset (tabulating the precise affinity across a library) is challenging to interpret: Given that a ligand is known to be bound, the exact binding location(s) within the ligand that contribute to its selection are unknown a priori.

To overcome these challenges, we used a closely coordinated computational and experimental strategy. On the computational side, we used the modeling framework of ProBound (Rube et al., 2022), which we here also refer to as free‐energy regression. In our previous study, which primarily focused on protein‐DNA interaction, we showed that ProBound can infer sequence‐to‐affinity models for hundreds of transcription factors from multi‐round selection data generated using fully random DNA libraries (Rube et al., 2022). In the same study, we also showed that ProBound can learn a model for predicting the enzymatic activity of the tyrosine kinase c‐Src as a function of the peptide sequence of the substrate from data generated using bacterial display of a random peptide library combined with pull‐down using a phospho‐specific antibody after exposure to the enzyme. In both cases, the predictions were valid over multiple orders of magnitude of affinity/activity. The purpose of the present study is to show that a similar strategy can be used to model the equilibrium binding specificity of non‐enzymatic protein‐peptide interactions.

Using experimental data for a specific peptide binding domain from the SH2 family, ProBound learns a model that can predict the binding free energy ∆∆G—relative to the optimal sequence, which is learned as part of the model fit—for any peptide sequence. In this study, we use the simplest such model, which assumes additivity of binding free energy over all residue positions in the peptide. We define relative binding affinity as $\exp\left(‐∆∆G/\mathrm{RT}\right)$, which equals one for the optimal sequence and takes values between zero and one for all other sequences; relative affinity is also inversely proportional to the equilibrium dissociation constant *K*_D_.

All possible binding offsets are being summed over when predicting enrichment in the ProBound model, which obviates the need to identify a discrete set of binding sites. To estimate the free‐energy parameters of the binding models, ProBound uses likelihood maximization. Specifically, given a pair of input and affinity‐selected libraries, the likelihood of the observed counts is computed based on the predicted affinities. If the input and selected libraries have been sequenced for multiple selection rounds, the likelihood can be computed separately for each round and then summed, allowing a single binding affinity model to be fit to multiple rounds, which integrates information about low‐ and high‐affinity sequences from the early and late rounds, respectively. Finally, because our regression approach does not require the initial library to be uniform or have large counts for individual sequences, we can explore how varying the degree of randomness in the initial library impacts the robustness of the final models and the degree of affinity selection required to get a sufficiently strong signal.

### 2.2 Robust inference of SH2‐peptide binding free energy models using ProBound

A recent study involving some of the authors of this work (Li et al., 2023) used bacterial surface display of plasmid‐encoded peptides containing a central phosphorylated tyrosine and NGS to assay the target specificity of the SH2 domain from c‐Src kinase. Two distinct library designs were used: one (“pTyrVar”) based on ~10^4^ peptides containing a phosphorylated tyrosine and occurring in the human population, the other a synthetic random library (“X_5_YX_5_”) with a fixed tyrosine between two fully degenerate five‐amino acid flanks with a theoretical diversity of ~10^13^ and an actual diversity of ~10^6^ (Figure 1a). Importantly, this study relied on position‐specific maps of relative enrichment, obtained by comparing amino‐acid frequencies directly before and after affinity‐based enrichment, to provide a simple and intuitive way to summarize the sequence preferences of the SH2 domain and predict relative binding affinities of unseen peptides (Li et al., 2023).

**FIGURE 2:**
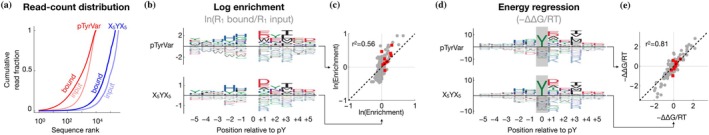
Comparison of amino‐acid enrichment analysis and free‐energy regression. (a) Distribution of read counts (after down‐sampling to 500,000 reads) for sequences in the pTyrVar and X_5_YX_5_ libraries, respectively, each before and after one round of affinity selection with the c‐Src SH2 domain. (b) Amino‐acid log‐enrichment due to affinity selection for c‐Src SH2, displayed as sequence logos, for the designed pTyrVar and random X_5_YX_5_ library, respectively. (c) Direct comparison of log‐enrichment parameters between the two library designs. Red points indicate tyrosine, all other residues are gray. (d) Inferred free‐energy contributions (ΔΔ*G*/RT) at different positions within the c‐Src SH2 binding interface, displayed as sequence logos. Gray rectangles indicate position where the model was constrained to recognize (phospho)tyrosine. (e) Direct comparison of ΔΔ*G/*RT parameters between the two library designs.

We hypothesized that using relative enrichment as a proxy for the true binding free energy differences (∆∆G/RT) associated with amino‐acid substitutions in the peptide could be suboptimal from a quantitative point of view: Relative enrichment may depend on library design, whereas binding free energy differences, being an intrinsic property of the SH2‐peptide interface, should not. To test this, we first compared peptide enrichments from the pTyrVar and X_5_YX_5_ libraries upon selection by the c‐Src SH2 domain. For each library design, the shift in the distribution of unique sequences by their count in the library (Figure 2a) indicates how the affinity‐based selection step boosts the higher‐affinity sequences at the expenses of lower‐affinity ones, causing a smaller number of sequences to dominate the bound library. The distributions for the pTyrVar library are to the left of those for the X_5_YX_5_ library, because pTyrVar is less complex than the fully random X_5_YX_5_ library. Note that amino acid preferences appear to be more pronounced for the pTyrVar library even though the screens were conducted under identical conditions (Figure 2b,c).

Our next goal was to assess whether ProBound (Rube et al., 2022) is also capable of building accurate sequence‐to‐affinity models from high‐throughput protein‐peptide binding data (Figure 1c). To this end, we configured ProBound to learn a free‐energy matrix that encodes how the SH2 domain interacts with an 11‐amino‐acid subsequence (Figure 1d). To focus this matrix on sequence‐specific SH2 binding, the central column was constrained to recognize tyrosine. The non‐central columns were learned using maximum likelihood estimation over the input and binding‐selected libraries. Specifically, the selection of each sequence was modeled by first computing the total sequence‐specific binding affinity (scoring and summing over all binding offsets, thus controlling for non‐central tyrosines) and then adding a non‐specific term to capture background selection and simple sequence biases (see “Methods”).

An advantage of our modeling approach is that it considers all offsets at which the ligands in the peptide library can be bound, meaning it can learn from data containing tyrosines at non‐central positions. We found that the ∆∆G/RT parameters in the resulting models were far more consistent between the two library designs (Figure 2d,e; *r* ^*2*^ = 0.81) than the corresponding log‐enrichments (Figure 2b,c; *r* ^2^ = 0.56); this is likely due to the fact that ProBound controls for sequence context and the effect of non‐specific binding, both of which are library dependent, when estimating the energetic effect of amino‐acid substitutions. The log‐enrichment analysis suggests selection for tyrosine at non‐central positions (added  2b,c), but this is likely an artifact since the libraries contain non‐central phosphotyrosine residues that may also be enzymatically phosphorylated, resulting in SH2 binding at non‐central offsets. By contrast, our ProBound models, which account for all possible binding registers, do not show disproportionate enrichment for tyrosine at non‐central positions (Figure 2d,e). Our model‐based analysis also demonstrates in a unbiased manner that a library design with five random positions on either side of the central tyrosine has a sufficiently large footprint to fully capture the relationship between sequence and affinity, and that the strongest effects are from positions −2 through +3 (Figure 2d).

### 2.3 Feasibility of using fully random libraries over multiple successive rounds of selection

**FIGURE 3:**
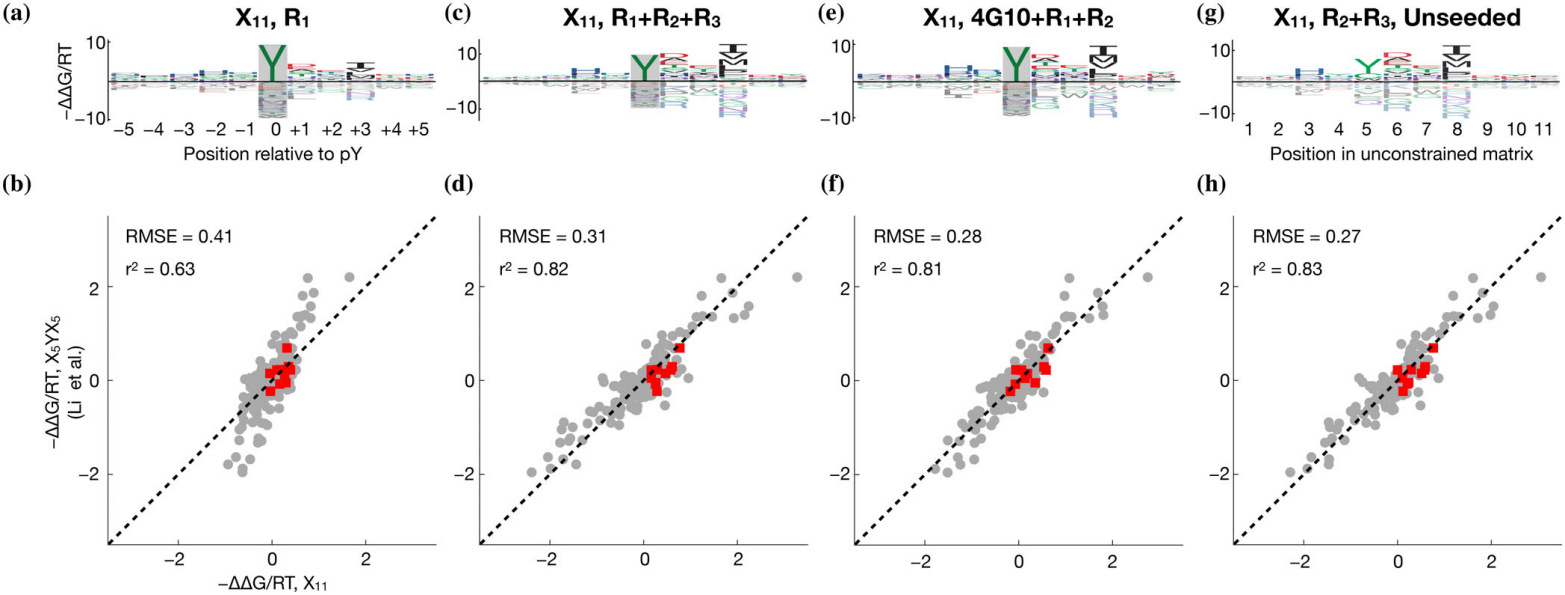
Multi‐round profiling of c‐Src SH2 using the naïve and pre‐enriched X_11_ libraries. (a) Binding model learned using one selection round and starting with the naïve X_11_ library. (b) Scatter plot comparing the model coefficients shown in panel (a) to the coefficients of the X_5_YX_5_ model shown in Figure 2d. Red points indicate tyrosine. (c), (d) Same as (a), (b) but showing a model that was trained on data from three selection rounds. (e), (f) Same as (a), (b) but showing a model that was trained on an experiment where the input library was pre‐selected using the 4G10 antibody, followed by two rounds of c‐Src SH2 binding selection. (g), (h) Same as (a), (b) but showing a model that was trained on data from the second and third selection rounds and that was not constrained to recognize tyrosine at the central position.

The library designs used so far exploited prior knowledge about the SH2 domains, specifically, their strong requirement for a phosphorylated central tyrosine residue at the binding interface (Waksman et al., 1992; Waksman et al., 1993). Such a biased design, however, may be undesirable or unfeasible when a similar experimental strategy is to be used to characterize other peptide binding domains. More generally, if it were feasible to generate suitable training data for ProBound using random libraries, which by definition would be universal and therefore could be used as the starting point for characterizing *any* peptide binding domain, this would obviate the need to design and synthesize libraries. We therefore repeated our peptide binding assay using a new library (“X_11_”) in which 11 consecutive residue positions are fully random; we otherwise followed the protocol of (Li et al., 2023), including phosphorylation of the bacterial display library before c‐Src SH2 domain binding. The results are shown in Figure 3a. Using ProBound to model the evolution of the X_11_ library over a single round (R_1_) of selection yielded binding free energy parameters much less consistent with those we obtained using the X_5_YX_5_ library (Figure 3b; *r* ^*2*^ = 0.63). Since X_11_ is expected to be more dominated by weak binders compared to the other two libraries, it was perhaps not surprising that the R_1_ data for X_11_ do not contain adequate signal for successful binding model inference. To address this, we developed a multi‐round selection strategy that aims to maximize recovery of the bound fraction of the library in each round (see “Methods”). In addition to the bound fraction, the input was also sequenced as a control for each round, and ProBound was configured to jointly learn from all input–output pairs across all rounds. Using this protocol to generate additional data, we found that three rounds of selection with the X_11_ library were necessary and sufficient to obtain adequate signal for building high‐quality sequence‐to‐affinity models for the c‐Src SH2 domain using ProBound (Figure 3c) that agreed well with the model built from R_1_ of the X_5_YX_5_ library (Figure 3d; *r* ^*2*^ = 0.82).

A disadvantage of using the unbiased X_11_ library to profile the binding preferences of SH2 domains is that many sequences in the input library will not contain any tyrosine residues (Figure S1). Moreover, depending on its flanking sequence, not every Tyr will be as efficiently phosphorylated by the kinases used for enzymatic phosphorylation. Because specific recognition of pTyr is a major contributor to SH2 binding affinity, we performed target‐agnostic pre‐selection on the X_11_ library using a biotinylated anti‐phosphotyrosine antibody (4G10) as described in (Li et al., 2023). We found that two subsequent rounds of c‐Src SH2 domain selection were sufficient in this case to obtain a high‐quality binding model (Figure 3e) that agrees well with the X_5_YX_5_ model (Figure 3f; *r* ^*2*^ = 0.81).

An advantage of the X_11_ library its universality: Even proteins with completely unknown sequence preferences could in principle be characterized in an unbiased manner given enough selection. We therefore asked if the c‐Src SH2 domain could be characterized without using the prior knowledge that having a tyrosine at the central position is required for binding. Reconfiguring ProBound to learn a completely unconstrained free energy matrix from the R_2_ and R_3_ libraries produced a binding model (Figure 3g,h) whose ∆∆G/RT parameters again agreed well with those of the centrally constrained model built from R_1_ of the X_5_YX_5_ library (*r* ^2^ = 0.83).

### 2.4 Quantifying differences in flanking sequence preference among paralogous SH2 domains

**FIGURE 4:**
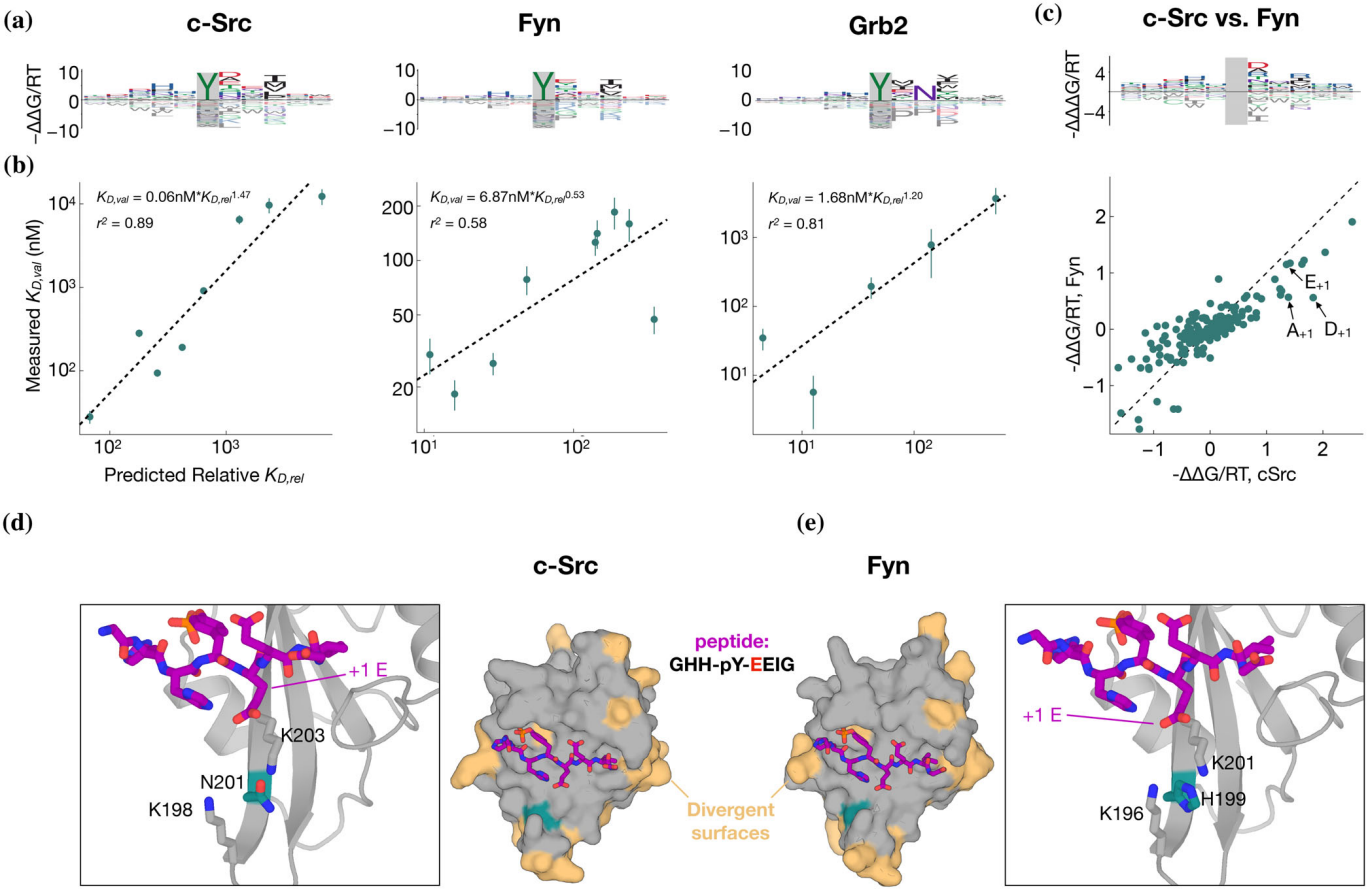
Flanking specificity of the c‐Src, Grb2 and Fyn SH2 domains. (a) Energy logos for the c‐Src SH2, Fyn SH2 and Grb2 SH2 binding models. (b) Scatter plots comparing the predictions from the binding models in (a) with competitive fluorescence polarization measurements. Vertical bars indicate standard error. Dashed black lines (and accompanying model expressions and *r* ^2^ values) indicate linear regression fits to the log‐transformed *K*_D_‐values. (**c**) Comparison of the c‐Src and Fyn binding models from (a) using an energy logo (top, showing the difference −∆∆∆G/RT between the model coefficients) and a scatter plot (bottom). (e), (d) AlphaFold 3 models of the c‐Src and Fyn SH2 domains (shown as surfaces in the central panels) bound to a high‐affinity phospho‐peptide (GHH‐pY‐EEIG, shown as purple sticks). Residues on the SH2 domains colored in beige are sites where c‐Src and Fyn diverge. A key divergent site (N201 in c‐Src and H199 in Fyn) is shown in teal. The zoom‐in panels highlight key residues in a cationic pocket on the SH2 domain that interacts with the ±1 residue on the peptide ligand.

To test the ability of our approach to resolve differences in binding specificity between paralogs, we performed two‐round selections of the X_5_YX_5_ library against the SH2 domains of two closely related kinases from the Src subfamily (c‐Src and Fyn) and of one distantly related SH2 domain from the adapter protein Grb2. For the Grb2 SH2 domain, we observed a strong preference for an asparagine (N) residue at position +2 relative to the pTyr (Figure 4a), consistent with previous findings (Songyang et al., 1993). Notably, for Grb2, the relative peptide binding affinities predicted by our model for peptides with and without an N_+2_ were separated by 2–3 orders of magnitude (Figure S2). This is consistent with previous measurements of Grb2 SH2 affinity, which indicate that substitution of the N_+2_ residue disrupts binding affinity by at least 100‐fold (Rahuel et al., 1996).

As a more stringent and direct experimental test of the predictive capability of our sequence‐to‐affinity models, we conducted low‐throughput competition fluorescence polarization assays for a panel of individually synthesized phosphopeptides derived from natural proteins, which covered almost two orders of magnitude of predicted binding affinity (see “Methods”). The measured ln(*K*_D_) values for each of the three SH2 domains showed good to excellent agreement with the corresponding ∆∆G/RT values predicted using our ProBound models (Figure 4b; Table S3; *r* ^2^ values ranging from 0.58 to 0.89; we confirmed that none of the validation sequences occurred in the raw NGS data on which our model was trained). For Grb2, we also directly compared our affinity predictions with spot intensities (Li et al., 2008) from a cellulose membrane array containing 720 defined peptides that was incubated with fluorescently labeled SH2 protein (Figure S3; *r* ^2^ = 0.51).

When comparing the c‐Src and Fyn models, we noted a few reproducible differences in specificity. For example, whereas both domains preferred a glutamic acid at the +1 position relative to the tyrosine (E_+1_), c‐Src had a distinctive preference for aspartic acid (D_+1_) and alanine (A_+1_) relative to Fyn (Figure 4c). To rationalize these differences, we used AlphaFold 3 (Abramson et al., 2024) to generate models of the c‐Src and Fyn SH2 domains bound to a predicted high‐affinity phosphopeptide bearing an E at the +1 position. The c‐Src and Fyn SH2 domains have 66% sequence identity, with very few divergent positions within the phosphopeptide binding pocket (Figure 4d,e). One such residue is N201 in c‐Src, which corresponds to H199 in Fyn. This residue sits at the center of a positively charged pocket that coordinates E_+1_ (Figure 4d,e). Our structural models and affinity models suggest that alteration of this central residue from an obligate neutral amino acid in c‐Src (N) to a potentially charged amino acid in Fyn (H) changes both the steric and electrostatic surface potential in this region, thereby altering amino acid preferences at peptide position +1.

Finally, in a more comprehensive test of the feasibility of using a target‐agnostic library approach, we also performed three rounds of selection of X_11_ against the same set of SH2 domains. Apart from one exception, the binding models inferred from the data we generated (Figure S5; Tables S1 and S2) cluster by SH2 domain identity (Figure S4). This indicates that ProBound is appropriately controlling for the significant differences between the X_11_ or X_5_YX_5_ library and whether 4G10 pre‐selection was used or not.

### 2.5 Affinity models enable the discovery of putative novel interactions

**FIGURE 5:**
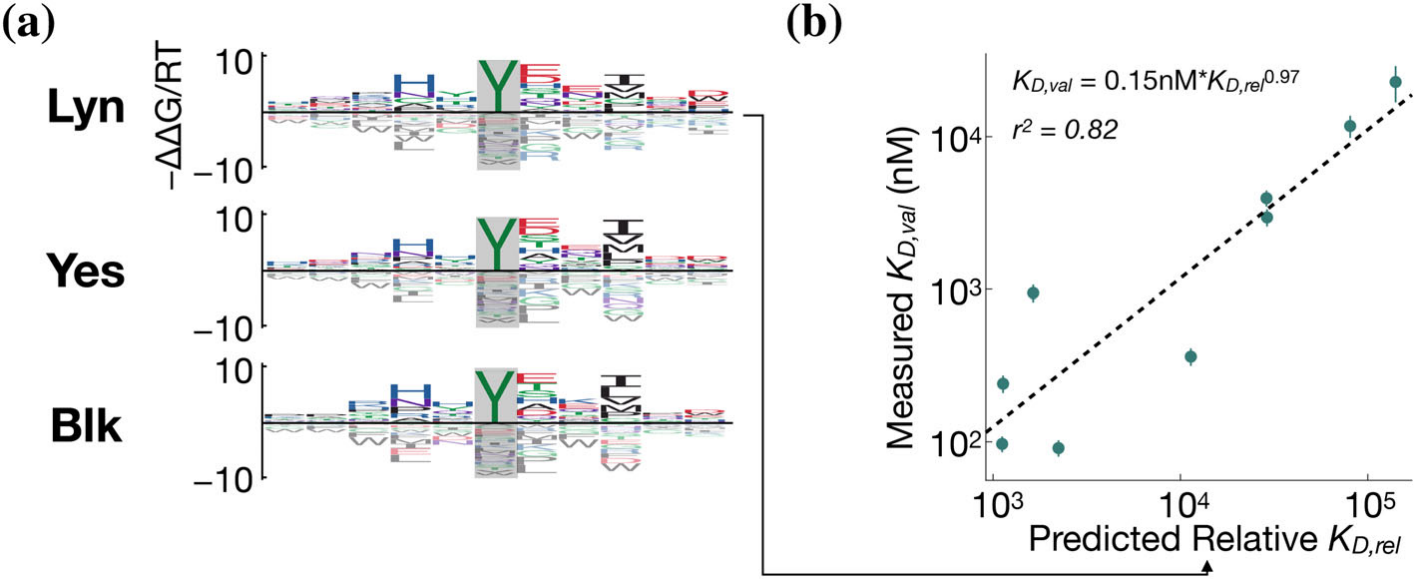
Flanking specificity for the Lyn, Yes and Blk SH2 domains. (a) Energy logos showing binding models for Lyn, Yes, and Blk. The models were trained on two‐round experiments using the X_5_YX_5_ starting library. (b) Scatter plots comparing model predictions and validation measurements for the Lyn SH2 domain, shown as in Figure 4b.

Having validated their accuracy experimentally, we sought to investigate whether our SH2 domain affinity models could be used to assign relative affinities to known phosphorylation sites in the human proteome, with the goal of identifying putative interaction partners not previously reported in the literature. First, we generated bacterial display datasets and affinity models for three additional SH2 domains from Src‐family kinases Lyn, Blk, and Yes (Figure 5a). For validation of the affinity model for Lyn, we selected a subset of phosphopeptides that were likely to fall within the dynamic range of the competitive fluorescence polarization assay (see “Methods” for details). We selected 9 phosphorylation sites that should bind to the Lyn SH2 domain with moderate to high affinity, including 3 previously reported interactors (PLC*γ*2 pY753, CSFR pY699, and SHP1 pY564) and 6 sites on proteins not previously reported to interact with Lyn (CD3ζ pY83, SLAP‐130 pY771 and pY595, TRAF3IP3 pY322, CD84 pY296, and SIGLEC5 pY544) in the STRING database (Szklarczyk et al., 2023). We measured binding affinities to the corresponding phosphopeptides by fluorescence polarization. The Lyn SH2 domain showed a good correlation between measured binding affinities and relative affinities predicted by the model (Figure 5b; *r* ^2^ = 0.82; again, none of the validation sequences occurred in the raw NGS data), indicating that this should be a reliable strategy to identify putative SH2 interactors across the proteome.

(Ronan et al., 2020) performed a reanalysis of the data from multiple medium‐throughput SH2‐pTyr interaction screens, resulting in a classification of a large number of phosphopeptides as bound or non‐bound. Figure S6 shows the distributions of relative affinities as predicted by our models for sequences classified as binding and non‐binding, respectively, by Ronan et al., for six different SH2 domains. The shift in distribution between binding and non‐binding sequences is in the expected direction for all six cases, and is statistically significant (*p* <0.05; Mann–Whitney U test) for 4 out of 6 cases.

We next used our models to predict relative affinities for all of the human tyrosine phosphorylation sites documented in the PhosphoSitePlus database (Hornbeck et al., 2015). While our models predict the potential for interaction regardless of context, such interactions will only be realized in a particular cellular context when a given SH2‐containing kinase and its target protein are both expressed. We therefore did not consider pairs that do not co‐express according to the STRING database (Szklarczyk et al., 2023). The lists of predicted high‐affinity binders for each SH2 domain (Table S4) show that the majority of the phosphorylation sites have low binding scores, indicating the preference of the SH2 domains toward a relatively small number of phosphorylation sites. It is noteworthy that none of the phosphorylated sites reach the maximal theoretical binding score, suggesting that few, if any, phosphosites in the proteome have evolved to be bound by their cognate SH2 domains at the highest possible binding affinity. This is consistent with earlier findings for SH2 domains (Haslam & Shields, 2012; Kaneko et al., 2012; Porter et al., 2000), and similar observations have been made for SH3 domains (Zarrinpar et al., 2003).

### 2.6 Affinity models correctly predict effects of single‐amino‐acid substitutions in SH2 ligands

The fact that SH2 domains display position‐specific sequence preferences means that single‐amino‐acid substitutions flanking the phosphorylated tyrosine could have a significant effect on SH2 binding affinity, and this should be captured by our ProBound models. To test this, we produced several pairs of phosphorylated peptides derived from human proteins, with one peptide in each pair a reported wild‐type sequence and the other containing a single‐amino‐acid mutation reported to be a natural human variant (Hornbeck et al., 2015) (Table S5). We experimentally measured binding affinities for these peptides against the c‐Src and Fyn SH2 domains and also predicted their relative affinities using the corresponding ProBound models. For both SH2 domains, the change in measured binding affinity due to the mutation had the same directionality as predicted by the affinity models (Figure S7), indicating significant qualitative predictive power (*p* = 0.0078, binomial test, *n* = 7). However, our validation set was too small to also claim quantitative predictive ability of our models for the variant effect.

**FIGURE 6:**
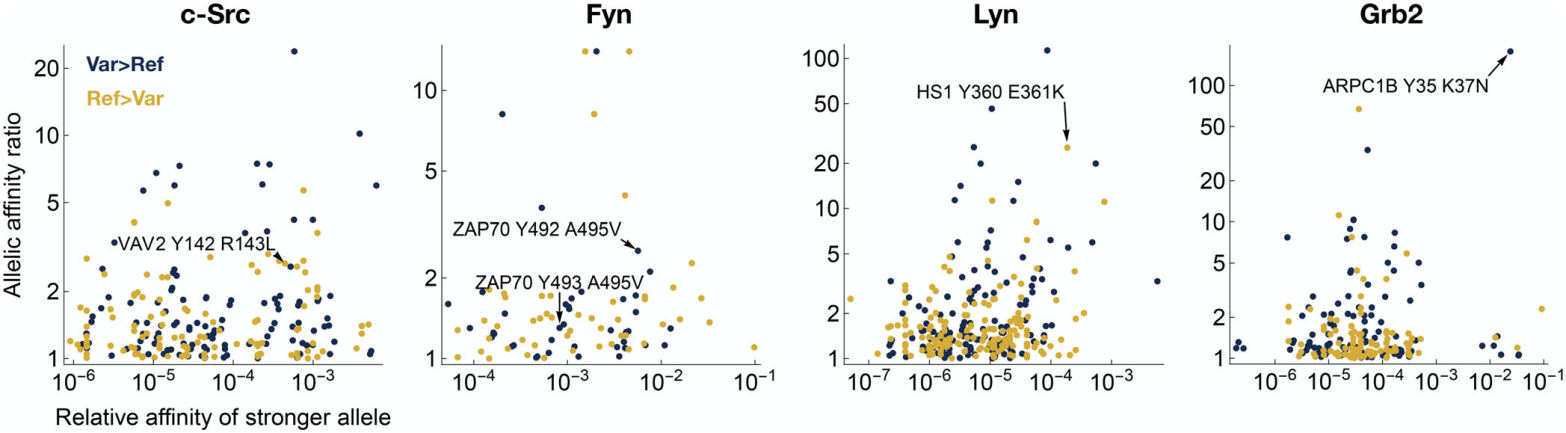
Distribution of the predicted quantitative impact of missense variants in SH2 binding sites in the human proteome. Scatterplot of allelic effect of missense variation in SH2 binding sites documented in the PTMVar database of human phosphorylation site variants (Hornbeck et al., 2015), colored by the direction of the effect. The *x*‐value corresponds to the greater of the predicted affinities of the two alleles, where relative affinity score is inversely proportional to the *K*_D_; the *y*‐value corresponds to the ratio of predicted affinities between the two alleles.

We next used the ProBound models to score all sequences in the PTMVar database of human phosphorylation site variants (Hornbeck et al., 2015), to predict if the reported variants enhance or diminish binding relative to the wild‐type sequence (Table S6). Notably, many of the variants in this database are derived from patients with specific disease phenotypes, and these mutations may contribute to rewired pathogenic signaling events. Filtering for proteins that co‐express with a given SH2 domain and removing variants with substitution of the phosphorylated tyrosine itself yielded the list of predicted variant effects summarized in Figure 6. Each dot denotes a variant corresponding to a pair of peptides differing by one point mutation. Each of these alleles can be scored in terms of a relative affinity whose reference is the highest‐affinity peptide for the same SH2 domain (for which relative affinity equals one by definition). The relative affinities thus predicted for each of the two alleles associated with a given variant can be compared by computing their ratio. A ratio equal to one means that the relative affinity (compared to the highest affinity peptide for the same SH2 domain) of both alleles is predicted to be the same (and therefore also their absolute *K*_D_).

For some of our predictions of a relatively large effect on SH2 binding affinity, we found literature evidence suggesting they might indeed provide a mechanistic explanation for an observed functional effect. For example, the c‐Src SH2 domain exhibits loss of affinity for Vav2 at the Y142 phosphorylation site in the presence of a lung cancer variant, R143L; c‐Src is directly involved in tyrosine phosphorylation at this same site in Vav2 (Servitja et al., 2003), and both proteins co‐localize with EGFR, a known driver of lung cancer. Our models also predict that the Fyn SH2 domain has an increased affinity for the Y492 phosphorylation site on ZAP‐70 in the presence of the A495V variant. Both ZAP‐70 and Fyn play a key role in mediating T cell activation downstream of the T cell antigen receptor, and Y492 and adjacent Y493 on ZAP‐70 are both are critical phospho‐regulatory sites in this pathway (Chan et al., 1995). For the Lyn SH2 domain, we predict a loss of affinity toward the HS‐1 Y360 phosphorylation site when the G361K variant is present. This is likely to have implications in the context of the Lyn/HS‐1 signaling axis, which is of therapeutic interest for Chronic Lymphocytic Leukemia (ten Hacken et al., 2013). Finally, for the Grb2 SH2 domain, we observed a strong enhancement in binding toward the Y35 site of ARPC1B with the K37N variant. Given the role of ARPC1B in actin cytoskeleton scaffolding, this neo‐interaction with Grb2, a ubiquitous adaptor protein, could alter the recruitment of other signaling proteins to the cytoskeleton during important cellular events, such as T cell recognition of antigen presenting cells, where both ARPC1B and Grb2 have already been functionally implicated (Dupre et al., 2021).

## 3 DISCUSSION

In this study, we have demonstrated how bacterial display of peptide libraries, multi‐round affinity‐based selection, and NGS can be combined with principled regression of binding free energy parameters to obtain robust models of peptide binding specificity. Our free‐energy regression approach has several advantages over simpler enrichment‐based strategies. First, when estimating the energetic impact of an amino acid substitution, the confounding impact of the other residues in the binding site is controlled for by predicting the affinity of the full binding interface, rather than simply computing the amino‐acid enrichment at each position independently. Similarly, the confounding impact of possible secondary binding sites is controlled for by considering all possible offsets and predicting the total binding affinity. Finally, data were synthesized by fitting a single binding model that best explains the full multi‐round experiment. Together, these improved computational methods enable an unbiased, target‐agnostic approach using highly complex random libraries.

Applying our methodology to various peptide binding domains from the SH2 family, we found that the predictive accuracy of the models depends strongly on the identity of the SH2 paralog, as well as on the design of the selection assay used to generate the training data. The presence of a post‐translationally modified phosphotyrosine in the center of the bound peptide, which contributes strongly and specifically to the binding affinity for all SH2 domains, may have contributed to the success of our current approach.

For each SH2 domain for which we generate NGS data and fit a model, we can predict binding affinity (relative to that of the highest‐affinity sequence) for *any* sequence flanking the pY. We found that it is important to objectively quantify the accuracy of each model by obtaining low‐throughput measurements of binding constants for a small set of synthesized peptides and directly comparing these to the model's predictions of relative affinity. However, once our sequence‐to‐affinity model for a particular SH2 domain passes this stringent quality assessment step, it is likely to give reliable predictions across the full theoretical space covered by the library on which it was trained. As such, it is a valuable tool for predicting putative new targets of the SH2 domain in the cell's protein–protein interaction network or for predicting the quantitative effect of allelic variation in the ligand sequence on SH2 binding affinity.

It is important to emphasize that the data on which we train our models does not include any direct affinity measurements for individual sequences. It consists exclusively of the sets of raw NGS reads sequenced in each round of our selection assay. Because the random libraries from which we start are highly diverse, and our simple additive model of binding free energy only has a few dozen parameters, the risk of overfitting to the training data is minimal. Nevertheless, we made sure that none of the sequences we used in our validation assays were present in the NGS training data.

The human genome encodes a great variety of PRDs (Tompa et al., 2014). To make progress in understanding the structure and function of the protein interaction networks defined by these domains, it will be essential to construct high‐quality sequence‐to‐affinity models for each of them and move beyond the binary classification in terms of targets and non‐targets that characterizes currently available resources (Kumar et al., 2024). The application of peptide display coupled with NGS should make it feasible to generate high‐throughput training data and perform unbiased analyses similar to what we did here for SH2 domains across the many other biologically important families of PRDs.

## 4 MATERIALS AND METHODS

### 4.1 Cloning, expression, and purification of SH2 domains

The human SH2 domains from Fyn (142–248), Lyn (121–228), Blk (116–222), and Yes (150–257) kinases were PCR‐amplified from plasmids encoding the full‐length proteins and cloned using the NEBuilder® HiFi DNA Assembly Master Mix (NEB) in a pET plasmid backbone that was PCR‐amplified with the primers pETXbaI_for and pETXhoI_rev (these and all other primers are defined in Table S7) from the pET‐His6‐SUMO‐Grb2(SH2), as reported before (Li et al., 2023), thus adding an N‐terminal His_6_‐SUMO tag and a C‐terminal AviTag® for biotinylation. The SH2 plasmids are available via Addgene: pET28‐His6‐SUMO‐Fyn‐SH2‐AviTag (214202), pET28‐His6‐SUMO‐Blk‐SH2‐AviTag (214204), pET28‐His6‐SUMO‐Lyn‐SH2‐AviTag (214207), pET28‐His6‐SUMO‐Yes‐SH2‐AviTag (214208), and pET28‐His6‐SUMO‐cSrc‐SH2‐AviTag (214230). For expression and parallel biotinylating of the SH2 domain product, *Escherichia coli* C43(DE3) cells carrying a BirA encoding helper plasmid were transformed with the respective SH2 domain expression construct and plated to grow overnight at 37°C on a streptomycin and kanamycin (100 μg/mL both) LB‐agar plate. The colonies were collected in 10 mL of TB medium and used to inoculate 1 L of TB medium and grown at 37°C and 215 rpm. Upon reaching an OD_600_ of 0.5, the expression of the SH2 domains was induced by adding IPTG up to 0.5 mM and biotin up to 250 μM, and the expressing culture was incubated at 18°C for 16–18 h. After removing the medium through centrifugation, the cell pellets were snap‐frozen in liquid N_2_ and stored at −80°C until purification. To purify the biotinylated SH2 domains, the cell pellets were resuspended in lysis buffer (50 mM Tris, pH 7.5, 300 mM NaCl, 20 mM imidazole, 10% glycerol), supplementing them with 2 mM *β*‐mercaptoethanol and 1× protease inhibitor cocktail before lysis by sonication (Fisherbrand Sonic Dismembrator). The soluble fraction separated after centrifugation at 33,000 g was run on a HisTrap HP column (Cytiva), then washed with 10 CV of the lysis buffer followed by 10 CV of wash buffer (50 mM Tris, pH 7.5, 50 mM NaCl, 10 mM imidazole, 10% glycerol). Next, the protein was eluted with elution buffer (50 mM Tris, pH 7.5, 50 mM NaCl, 250 mM imidazole, 10% glycerol) directly onto a 5 mL HiTrap Q HP anion exchange column (Cytiva). The biotinylated SH2 domain was then eluted from the anion exchange column with an NaCl gradient (50 mM Tris, pH 7.5, 0.05–1 M NaCl, 1 mM TCEP‐HCl), and the His_6_‐SUMO tag was cleaved using 0.05 mg/mL Ulp1 protease. The biotinylated SH2 domain was then collected in a flowthrough after running the cleavage reaction over a HisTrap HP column. Finally, the biotinylated SH2 domain in the flowthrough was run on a HiLoad 16/600 Superdex 75 pg size exclusion column (Cytiva) in storage buffer (20 mM HEPES, pH 7.5, 150 mM NaCl, 10% glycerol). The purified biotinylated SH2 domains were aliquoted, snap‐frozen in liquid N_2_, and stored at −80°C.

### 4.2 Expression and purification of kinases

The expression constructs for the four kinase domains EPHB1 (pET23a‐H6‐TEV‐EPHB1; AddGene: 79694), c‐Src (pET23a‐H6‐TEV‐cSrc; AddGene: 214233), Abl (pET23a‐H6‐TEV‐cAbl; AddGene: 214234), and AncSZ (pET23a‐H6‐TEV‐AncSZ; AddGene: 214235) were used to express and purify the four kinase domains, same as in reported before (Li et al., 2023). In short, *E. coli* BL21(DE3) cells with the YopH tyrosine phosphatase‐expressing helper plasmid were transformed with the constructs and plated on ampicillin and streptomycin LB‐agar plates. A single colony was used to grow an overnight starter culture that was used to inoculate 6 L of TB medium. The expression was induced by 0.5 mM IPTG at OD_600_ of 0.5 for 16–18 h at 18°C. The cells were harvested, resuspended in lysis buffer (50 mM Tris, pH 8.0, 300 mM NaCl, 20 mM imidazole, 10% glycerol), and captured on a HisTrap HP column. The kinases were eluted from the HisTrap column with high imidazole directly onto a 5 mL HiTrap Q HP anion exchange column. Next, the kinases were eluted from the HiTrap Q HP column with an NaCl gradient. The AncSZ kinase was then run in a final step on a HiLoad 16/600 Superdex 75 pg column (Cytiva) using the size exclusion and storage buffer (10 mM HEPES, pH 7.5, 100 mM NaCl, 1 mM TCEP, 5 mM MgCl2, 10% glycerol). For the other three kinases, the His_6_‐tag was then cleaved using TEV protease, and the cleaved kinase domains were isolated after flowthrough through a HisTrap HP column. The kinases were then purified by size exclusion chromatography on a HiLoad 16/600 Superdex 75 pg. column (Cytiva) using the size exclusion and storage buffer. Purified kinases were aliquoted, snap‐frozen in liquid N_2_, and stored at −80°C.

### 4.3 Cloning of the X11 library

The X_11_ and X_8_ libraries used in this investigation were prepared similarly to the already available X_5_YX_5_ library reported previously (Li et al., 2023). In all cases, the library is cloned at the N‐terminus of the eCPX that contains a Myc‐tag at the C‐terminus. For this purpose, the eCPX‐cMyc was PCR‐amplified from the reported pBAD33‐X_5_YX_5_‐eCPX‐cMyc‐tag plasmid library using the link‐eCPX‐fwd and eCPX‐rev primers. The NNS‐encoded oligonucleotide library with the 5′ SfiI restriction site and 3′ eCPX‐linker overlapping sequence obtained from MilliporeSigma, eCPX‐rand‐lib‐X11, was used as forward primer in combination with the eCPX‐rev reverse primer to produce the X_11_‐eCPX‐cMyc‐tag DNA library by PCR amplification. In parallel, the pBAD33 plasmid backbone was PCR‐amplified using the eCPX‐BB‐for and eCPX‐BB‐rev. The pBAD33 PCR‐amplified plasmid backbone was also treated with DpnI (NEB). Both the pBAD33 plasmid backbone and the eCPX‐appended libraries were treated with the SfiI DNA restriction enzyme (NEB) after PCR purification. The SfiI‐treated pBAD33 plasmid backbone was additionally treated with Quick CIP (NEB) as well. Both the DNA libraries and the pBAD33 plasmid backbone were then gel‐purified and used for a ligation reaction in a 5:1 molar ratio using the T4 DNA ligase (NEB). After PCR purification of the ligation, the DNA was used to transform NEB 10‐beta electrocompetent *E. coli* (NEB) using the Bio‐Rad xCell electroporator (Bio‐Rad) at 2 kV, 25 μF, and 200 Ω. After a 1 h recovery at 37°C in 1 mL of the recovery medium supplied with the electrocompetent *E. coli*, the transformed cells were used to inoculate 200 mL of LB containing 25 μg/mL chloramphenicol and grow a midi‐preparation culture overnight at 37°C and 215 rpm. The pBAD33‐X_11_‐eCPX‐cMyc and the pBAD33‐X_8_‐eCPX‐Myc plasmid libraries were then midi‐prepped from the overnight cultures (ZymoPURE™ II Plasmid Midiprep Kit).

### 4.4 Preparation of cells for display of phosphorylated peptide libraries

Bacterial display and phosphorylation of the displayed peptides was performed similarly to (Li et al., 2023), with small adjustments. In short, 50–100 ng of the X_5_YX_5_, X_11_, or X_8_ plasmid library was used to transform 25 μL of electrocompetent MC1061 F^−^ *E. coli* (Lucigen ≥4 × 10^10^ cfu/μg) in a 1 mm electroporation cuvette and micropulser (Bio‐Rad) set at 1.8 kV. The transformed cells were recovered for 1 h at 37°C and 215 rpm in 975 μL of warm recovery medium supplied with the electrocompetent cells. For the display culture, 100 mL of LB containing 25 μg/mL chloramphenicol were inoculated with 0.98 mL of the transformed cells after recovery and grown at 37°C and 215 rpm until reaching an OD_600_ of 0.5–0.6. At this point, 20 mL of the culture were induced with 0.4% (w/v) arabinose for 4 h (± 30 min) at 25°C and 215 rpm. Cells from 5 mL of the induced culture were collected by centrifugation at 4000 *g*, resuspended in 5 mL of PBS, and separated into several 750 μL aliquots. The cells of each aliquot were collected by centrifugation (4000 *g*), the supernatant was discarded, and cells were stored overnight at 4°C. For peptide phosphorylation, each cell pellet from a 750 μL aliquot was resuspended in 500 μL of kinase buffer (pH 7.5, 50 mM Tris, 10 mM MgCl2, 150 mM NaCl, 2 mM Orthovanadate, and 1 mM TCEP) and the volume of cells needed for the given experiments was used for phosphorylation of the displayed peptides by adding purified kinase domains c‐Src, c‐Abl, EPHB1, and AncSZ up to 2.5 μM, 5 mM of creatine phosphate, 50 μg/mL creatine phosphokinase (from rabbit muscle, Sigma), and 1 mM ATP. The phosphorylation reaction was incubated at 37°C for 3 h and stopped by adding EDTA up to 25 mM. The cells of the phosphorylated bacterial display library were then collected by centrifugation (4000 *g*) and resuspended in the same volume of the buffer used for the selection.

### 4.5 Multi‐round selection against SH2 domains

To perform the single selection experiment using the phosphorylated peptide library against, 75 μL of streptavidin‐coated magnetic beads (Dynabeads™ FlowComp™ Flexi Kit, Thermo‐Fisher) were washed twice in 1 mL of SH2 binding buffer (50 mM HEPES pH 7.5, 150 mM NaCl, 0.05% Tween 20, 1 mM TCEP) and incubated in a total of 150 μL SH2 binding buffer containing 20 μM biotinylated SH2 domain on a rotator at 4°C for 2–3 h in low protein‐binding microcentrifuge tubes (1.5 mL, Thermo Scientific™). The functionalized magnetic beads were then washed twice in 1 mL of SH2 binding buffer, resuspended in 75 μL of the SH2 binding buffer, and mixed with phosphorylated peptide‐displaying cells resuspended in 100 μL of the same buffer containing 0.05% Tween 20. After incubation for 1 h on a rotator at 4°C in the low protein‐binding microcentrifuge tubes (1.5 mL, Thermo Scientific™), the supernatant containing the non‐bound library fraction was discarded, and the magnetic beads with the bound fraction were washed in 1 mL of the SH2 binding buffer containing Tween 20 for 30 min on a rotator at 4°C. At this point, the magnetic beads with the bound fraction were collected, resuspended in 100 μL of MilliQ water, and incubated at 100°C for 10 minutes to extract the plasmid DNA. As the input library, 100 μL of the surface‐displayed library before phosphorylation was washed with MilliQ water through centrifugation, and the DNA was extracted the same as the selection sample. The extracted DNA was then used both for NGS sample preparation and for PCR amplification using the 3'SfiIX11‐fwd and eCPX‐rev primers to clone the enriched library in the pBAD33 plasmid backbone, as in the original libraries. The purified ligation was directly used for cell transformation and production of peptide‐displaying cells for the next round of selection.

### 4.6 Display and phosphorylation quality control

For every set of experiments, a new batch of phosphorylated peptide‐displaying cells was produced. A sample from each batch of these phosphorylated peptide‐displaying cells was used to control the quality and consistency by selection using an antibody that specifically binds to phosphorylated tyrosine, irrespective of the flanking sequence context (Li et al., 2023). For this quality control, 50 μL of the bacterial peptide library after phosphorylation was pelleted by centrifugation at 3000 *g* and resuspended in 50 μL PBS‐BSA buffer (PBS + 0.2% BSA) containing 1:1000 Platinum *α*‐PhosTyr 4G10 biotin conjugated antibody (Sigma). The cells were incubated with the antibody on ice for 1 h and then pelleted through centrifugation at 3000 *g* to remove the non‐bound antibody. In the same manner, the cells were washed once in 100 μL in PBS‐BSA, resuspended in 50 μL PBS‐BSA, and mixed with 37.5 μL of streptavidin coated magnetic beads (Dynabeads™ FlowComp™ Flexi Kit, Thermo‐Fisher) previously washed and resuspended in PBS‐BSA. The cell‐bead suspension was incubated on a rotator at 4°C for 20 min to capture the library fraction labeled with the biotinylated antibody. After removing the non‐bound fraction via magnetic separation, followed by a 15 min wash in 0.5 mL in PBS‐BSA, the plasmid DNA of the bead‐bound fraction of the surface‐displayed library was extracted by incubation at 100°C for 10 min in 50 μL of MilliQ water. The sample was then analyzed by NGS.

### 4.7 NGS and preparation of selection samples

DNA extracted from both the collected input and selection‐enriched samples was analyzed by NGS. Each sample was prepared for sequencing similarly as reported before (Li et al., 2023). In short, the DNA samples were first PCR‐amplified over 15 cycles using the Q5 polymerase master mix (NEB) and the primers altTruSeq‐eCPX‐fwd and altTruSeq‐eCPX‐rev. After analyzing small amounts of these PCR reactions on a DNA‐agarose gel, the PCR products were used for the second PCR amplification without any purification and roughly amount adjusted to the intensity of the bands in the control DNA gel, in order to normalize the input DNA levels. Sample‐specific indexing primer pairs (from the D500 and D700 primer series, see Table S7) were used in this second PCR to label the samples. The second‐round PCR products were then gel‐purified and quantified on NanoDrop so that equal amounts of each sample were mixed together. The final sample mix, constituted to yield around 1 million reads for each sample, was quantified by qPCR using the NEBNext Library quantification kit (NEB) and the StepOnePlus cycler (Applied Biosystems) to produce the needed DNA concentration of 4 nM. Before sequencing on the Illumina MiSeq system using the MiSeq Reagent Kit v3 (150 cycle), the sample was denatured in 0.1 M NaOH for 5 min at room temperature and diluted down to 20 pM. The quality of each sequencing run was controlled by adding up to 5% of denatured and diluted down to 20 pM PhiX (Illumina). Demultiplexing of the data by sample using the defined index pairs was performed automatically by Illumina BaseSpace.

### 4.8 Measuring affinities of SH2 domains and phosphopeptides using competitive fluorescence polarization

Measured binding constants (*K*_D_) from competition mode fluorescence polarization experiments were obtained using the purified SH2 domains used for the selection and chosen sets of peptides with a phosphorylated tyrosine exactly as described previously (Li et al., 2023). For all Src‐family kinase‐derived SH2 domains, the fluorescently labeled peptide FITC‐Ahx‐GDG(pY)EEISPLLL (c‐Src SH2 consensus) was used as the probe, and for the Grb2 SH2 domain, the fluorescently labeled peptide FITC‐Ahx‐FDDPS(pY)VNVQN (Ahx = 6‐aminohexanoic acid) from the Y177 phospho‐tyrosine site of BCR. Before performing the competition mode fluorescence polarization measurement, the *K*_D_ of the probes for the respective SH2 domains was first measured in a direct mode of fluorescence polarization. The sets of non‐labeled peptides sequences for the competition‐based measurement were chosen in different ways: For the Grb2 and Fyn SH2 domains, the choice was based on sequences found during selections against these SH2 domains, so that they span a larger range of predicted relative affinities. For the set of peptides where single point mutations were compared, phosphopeptides reported in the PhosphoSitePlus database (Hornbeck et al., 2015) and previously shown to bind to the c‐Src SH2 domain (Li et al., 2023) were chosen alongside their respective single point mutants. To choose phosphopeptides for validation of the Lyn SH2 domain, we started from all verified phosphopeptides found in PhosphoSitePlus, filtered based on their co‐expression with the Lyn kinase using the STRING database. Using the validation data for the Fyn SH2 domain, the range of predicted relative binding affinities that corresponds to the range between 1 nM and 100 μM, which is the dynamic range of reliable measurement of absolute binding affinities using our competitive fluorescence polarization setup, was defined and used to further filter the set of candidate phosphopeptides. A subset that evenly covers the chosen range of predicted relative affinities was finally picked for the validation measurements of the Lyn SH2 domain. For the c‐Src, Yes, and Blk SH2 domains, a combination of the previously chosen peptides was used, after verifying that their predicted relative affinities for the given SH2 domain fell within the same measurable range. All peptides containing phosphorylated tyrosine, both fluorescently labeled and non‐labeled, were produced by SynPeptide (China).

### 4.9 Processing of sequencing reads

For each sequencing library, the read pairs were first joined using FLASH‐1.2.11 software package (Magoc & Salzberg, 2011), and the combined reads were scanned for a 33‐bp region flanked by GTAGCTGGCCAGTCTGGCCAG to the left and GGAGGGCAGTCTGGGCAGTC to the right, allowing for up to five mismatches. After cropping out the flanking sequences, the remaining reads were filtered for exact matches to the library design, whereupon reads with a PHRED score below 20 at any position were discarded. The remaining reads were then translated to protein code. Count tables containing the number of occurrences of each sequence in the relevant input and bound libraries were finally created for each selection round of each experiment.

### 4.10 Fitting binding models

To learn binding models based on sequencing data, ProBound minimizes a loss function consisting of a log‐likelihood term and a regularization term:

$$l_{\text{total}}=l_{\text{data}}+l_{\mathrm{reg}}$$

ProBound was thus configured to load one or more of count tables (one per selection round), model each table as a single‐round SELEX experiment, and then compute the scaled binomial log‐likelihood:

$$l_{\text{data}}=\begin{array}{l} \sum_{c=1}^{N_{C}}\frac{1}{k_{c}}\sum_{\mathrm{i\epsilon}S_{c}}(k_{c,i,I}\ln\frac{\eta_{c,I}}{\eta_{c,I}+\eta_{c,B}\kappa_{B}\left(s_{i}\right)}\\ +\;k_{e,i,B}\ln\frac{\eta_{c,B}\kappa_{c}\left(s_{i}\right)}{\eta_{c,I}+\eta_{c,B}\kappa_{B}\left(s_{i}\right)}) \end{array}$$

Here $N_{C}$ is the number of count tables, $k_{c}$ is the total number of reads and S_c_ the set of unique sequences associated with count table c, $k_{c,i,I}$ and $k_{c,i,B}$ are the number of reads observed for sequence $s_{i}$ in the input and bound columns of count table c, and $\eta_{c,I}$ and $\eta_{c,B}$ are parameters controlling the predicted sequencing depth. $\kappa_{c}\left(s_{i}\right)$ is the predicted enrichment of sequence $s_{i}$ in the bound library. ProBound was configured to model this enrichment using two “binding mode” terms, one representing non‐specific binding and experimental biases (NS) and one term representing sequence‐specific SH2‐domain binding (S):

$$\kappa_{c}\left(s_{i}\right)=\begin{array}{l} \alpha_{c,\mathrm{NS}}\sum_{x=1}^{L-w_{\mathrm{NS}}+1}e^{\vec{X}\left(s_{i,x:x+w_{\mathrm{NS}}}\right)\cdot {\vec{\beta }}_{\mathrm{NS}}+\gamma_{x}}\\ +\;\alpha_{c,S}\sum_{x=1-f_{S}}^{L+f_{S}-w_{S}+1}e^{\vec{X}\left(s_{i,x:x+w_{S}}\right)\cdot {\vec{\beta }}_{S}} \end{array}$$

These terms are similar in that each receives additive contributions from all offsets along the sequence, and each contribution depends log‐linearly on the subsequence starting at the offset in question. Mathematically, $\vec{X}\left(s_{i,x:x+w}\right)$ is a predictor vector that uses one‐hot encoding to represent the subsequence $s_{i,x:x+w}$ starting offset x and extending w residues to the right. The coefficient vectors (${\vec{\beta }}_{\mathrm{NS}}$ and ${\vec{\beta }}_{S}$) encode the log‐fold effects that the residues in the substring have one the binding ($e^{\vec{X}\cdot {\vec{\beta }}_{\mathrm{NS}}}$ and $e^{\vec{X}\cdot {\vec{\beta }}_{S}}$). The components in ${\vec{\beta }}_{S}$ thus correspond to the entries in the free‐energy matrix −∆∆G/RT that we wish to learn. The overall scale of the selection was set by the “activity” weights $\alpha_{c,\mathrm{NS}}$ and $\alpha_{c,S}$, which took independent values for each count table c.

Beyond the similarities discussed above, the binding models were configured to differ in important ways. For the non‐specific mode to focus on simple sequence dependences, short subsequences of length $w_{\mathrm{NS}}=3$ were used (for Grb2, $w_{\mathrm{NS}}=1$ was used since longer substrings allowed this mode to capture the main motif YXNX. This was also done for the pTyrVar library). A position‐specific bias term $\gamma_{x}$ was also included to let this mode absorb non‐homogenous sequence biases. In contrast, the sequence‐specific binding mode was focused on extended SH2 binding sites by using substring of length $w_{S}=11$ and by not including a position‐specific bias term. Moreover, because the variable region in the library (which had length L=11) was flanked by the constant sequences GQSGQ on the left and GGQSG on the right, and because these sequences in principle could be a part of a binding site, the sum over offsets x was extened to include substrings overlapping up to $f_{S}=5$ flanking residues. Finally, to focus this mode on phosphotyrosine‐specific binding, the coefficient vector ${\vec{\beta }}_{S}$ was constrained to assign weight zero to a tyrosine at the central position and weight −10 for all other residues. The regularization term $l_{\mathrm{reg}}$ consisted of an $L_{2}$ regularizer, an exponential barrier term, and a Dirichlet term (with count 5) as described in the original publication (Rube et al., 2022).

## AUTHOR CONTRIBUTIONS

**Dejan Gagoski:** Conceptualization; writing – review and editing; methodology; investigation; writing – original draft; data curation. **H. Tomas Rube:** Conceptualization; writing – review and editing; methodology; investigation; writing – original draft; data curation; formal analysis; software. **Chaitanya Rastogi:** Conceptualization; writing – review and editing; funding acquisition; methodology; formal analysis. **Lucas A. N. Melo:** Writing – review and editing; methodology; formal analysis. **Xiaoting Li:** Investigation; writing – review and editing. **Rashmi Voleti:** Investigation. **Neel H. Shah:** Conceptualization; writing – review and editing; funding acquisition; supervision; project administration; writing – original draft; formal analysis. **Harmen J. Bussemaker:** Conceptualization; writing – review and editing; funding acquisition; supervision; project administration; writing – original draft.

## CONFLICT OF INTEREST STATEMENT

HTR, CR, and HJB are co‐inventors on a patent application (PCT/US2020/023017) related to the ProBound algorithm used in this study, and shareholders of Metric Biotechnologies, Inc.

## Supporting information


**Figure S1:** Proportion of sequences containing zero, one, or two or more tyrosine residues in the different input libraries.

**Figure S2:** Impact of N_+2_ on predicted Grb2 binding. Plot shows the distribution over the set of sequences represented in the X_5_YX_5_ library (shown using a log‐scale kernel density estimator) of binding affinities predicted by the Grb2 model in Figure 4a. Sequences containing an N_+2_ (blue) are grouped separately from the other sequences (orange).

**Figure S3:** Comparison of model predictions and published peptide array measurements for Grb2. Each point represents one of the 720 defined peptides on the cellulose membrane array that was incubated with fluorescently labeled SH2 protein by (Li et al., 2008). Predictions were computed using the Grb2 model shown in Figure 4a after truncating the left‐ and right‐most positions to align it with the nine‐residue sequences of the array.

**Figure S4:** Binding models for the c‐Src, Grb2 and Fyn SH2 domains. The models were learned using different combinations of starting libraries (X_5_YX_5_ or X_11_), selection round (R_1_ or R_1_ + R_2_), and replicates (rep 1 or rep 2).

**Figure S5:** Broader comparison of multi‐round models for multiple SH2 domains. The dendrogram shows the clustering of various binding models for the c‐Src, Grb2, and Fyn SH2 domains, built using ProBound from data generated using different starting libraries, number of selection rounds. Numbers in parentheses denote replicates. Arrows denote the X_5_YX_5_ models used for all other analyses in this paper.

**Figure S6:** Comparison of model predictions and previous classifications of phosphopeptides as binders and non‐binders. For each SH2 domain, the phosphopeptides classified by (Ronan et al., 2020) were scored using the models in Figures 4a and 5a and the empirical cumulative distribution functions of the resulting relative K_D_‐values were plotted separately for sequences classified as binders (yellow) and non‐binders (blue).

**Figure S7:** Impact of single‐amino‐acid substitutions on c‐Src and Fyn SH2 binding. The bar charts show the measured K_D_ value (left) and the predicted relative K_D_ (right) for pairs of naturally occurring sequence variants (highlighted letters). The predictions were made using the models shown in Figure 4a.

**Table S1:** Next‐generation sequencing datasets generated.
**Table S2:** List of ProBound analyses, settings, and resulting binding models.
**Table S3:** Validation measurements, unpaired.
**Table S4:** Predictions for peptides in the PhosphoSitePlus database, for cSrc, Fyn, Lyn, Grb2.
**Table S5:** Validation measurements, single‐amino‐acid substitution pairs.
**Table S6:** Predictions for peptides in the PTMVar database, for cSrc, Fyn, Lyn, Grb2.
**Table S7:** Cloning and sequencing primers used.

## Data Availability

The raw sequencing data, processed count tables, configuration files, AlphaFold 3 models, and two supplemental tables are available at datadryad.org/dataset/doi:10.5061/dryad.msbcc2g7w. ProBound models were fit using source code available at github.com/RubeGroup/ProBound. For each of the six SH2 domains that we built models for, relative affinities can be predicted from protein sequence using the JSON scoring models in Table S2 and a scoring script available at https://github.com/RubeGroup/2025_SH2_scoring.
