# Supplementary figures and images for: Accurate affinity models for SH2 domains from peptide binding assays and free‐energy regression
Source: Protein Sci. 2025 Oct 14;34(11):e70317. doi: 10.1002/pro.70317 (PMC12521615; doi:10.1002/pro.70317)

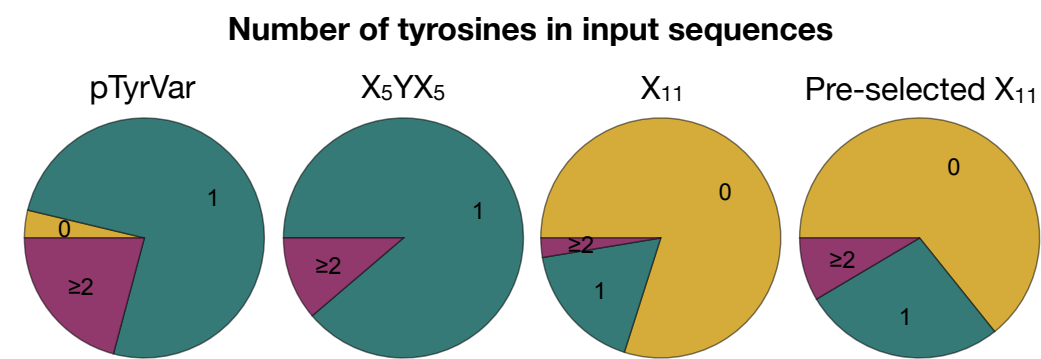

Supplement: Supplementary file 1 — Figure S1: Proportion of sequences containing zero, one, or two or more tyrosine residues in the different input libraries. [file PRO-34-e70317-s002.pdf]

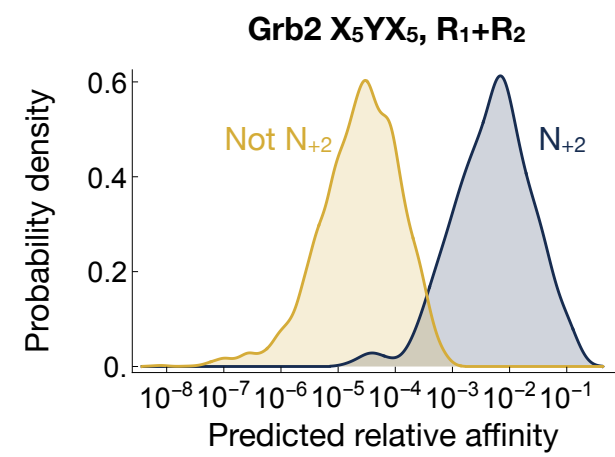

Supplement: Supplementary file 2 — Figure S2: Impact of N+2 on predicted Grb2 binding. Plot shows the distribution over the set of sequences represented in the X5YX5 library (shown using a log‐scale kernel density estimator) of binding affinities predicted by the Grb2 model in Figure 4a. Sequences containing an N+2 (blue) are grouped separately from the other sequences (orange). [file PRO-34-e70317-s006.pdf]

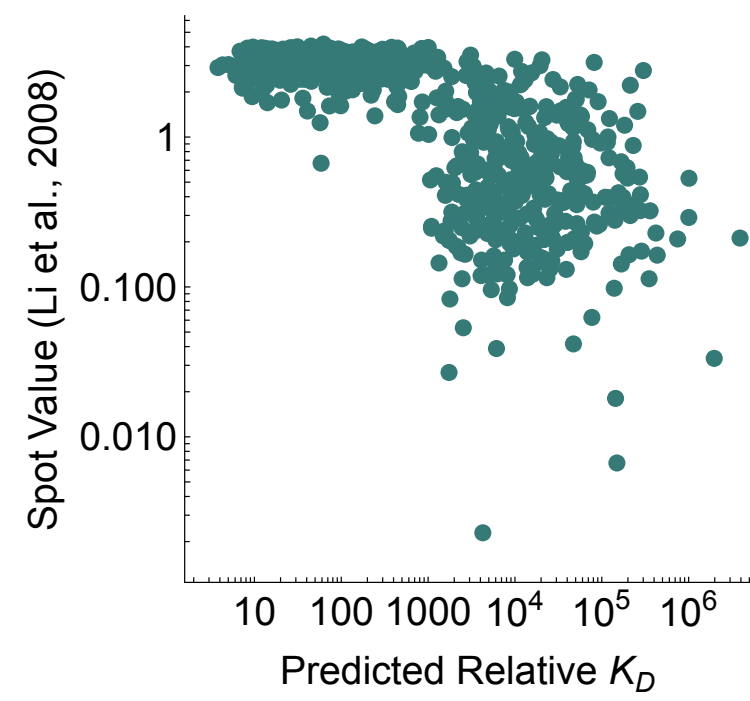

Supplement: Supplementary file 3 — Figure S3: Comparison of model predictions and published peptide array measurements for Grb2. Each point represents one of the 720 defined peptides on the cellulose membrane array that was incubated with fluorescently labeled SH2 protein by (Li et al., 2008). Predictions were computed using the Grb2 model shown in Figure 4a after truncating the left‐ and right‐most positions to align it with the nine‐residue sequences of the array. [file PRO-34-e70317-s003.pdf]

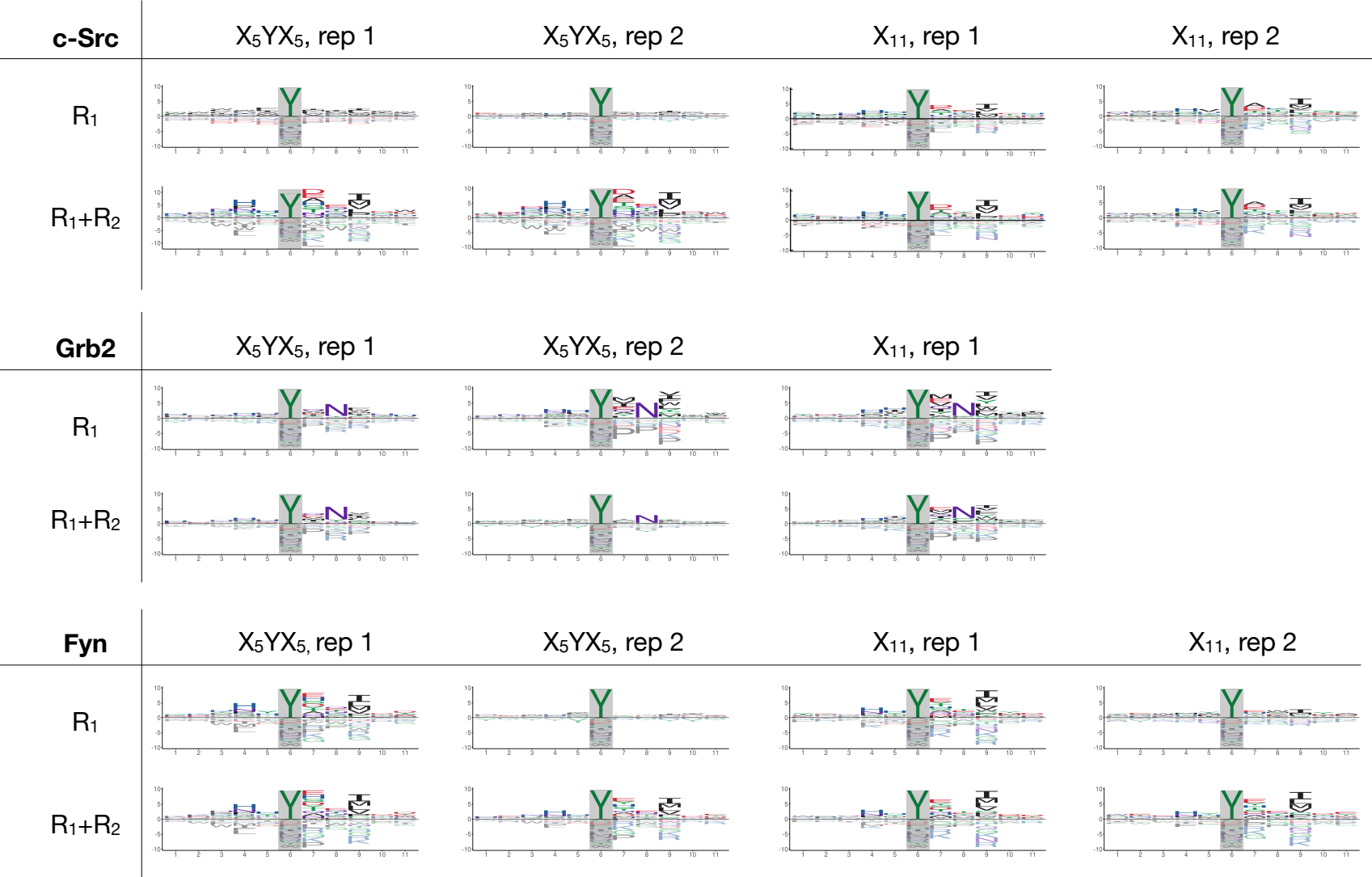

Supplement: Supplementary file 4 — Figure S4: Binding models for the c‐Src, Grb2 and Fyn SH2 domains. The models were learned using different combinations of starting libraries (X5YX5 or X11), selection round (R1 or R1 + R2), and replicates (rep 1 or rep 2). [file PRO-34-e70317-s001.pdf]

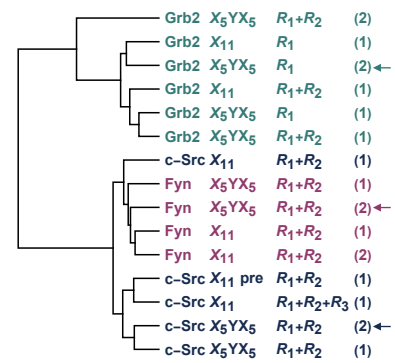

Supplement: Supplementary file 5 — Figure S5: Broader comparison of multi‐round models for multiple SH2 domains. The dendrogram shows the clustering of various binding models for the c‐Src, Grb2, and Fyn SH2 domains, built using ProBound from data generated using different starting libraries, number of selection rounds. Numbers in parentheses denote replicates. Arrows denote the X5YX5 models used for all other analyses in this paper. [file PRO-34-e70317-s004.pdf]

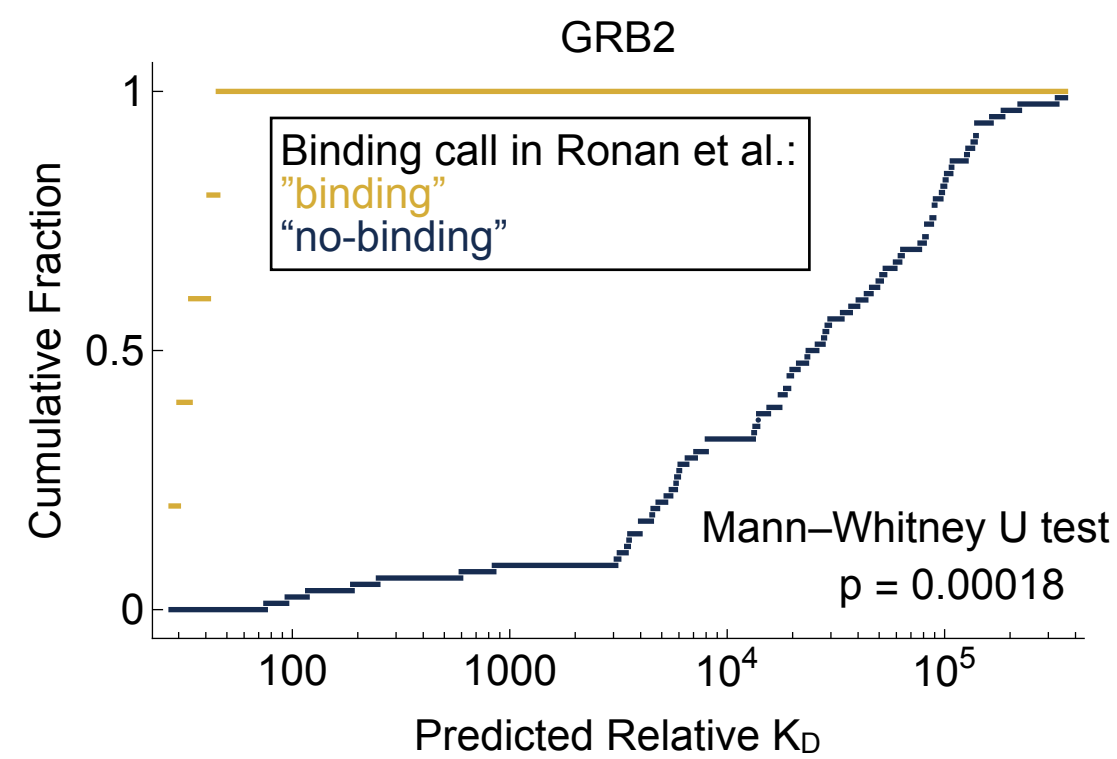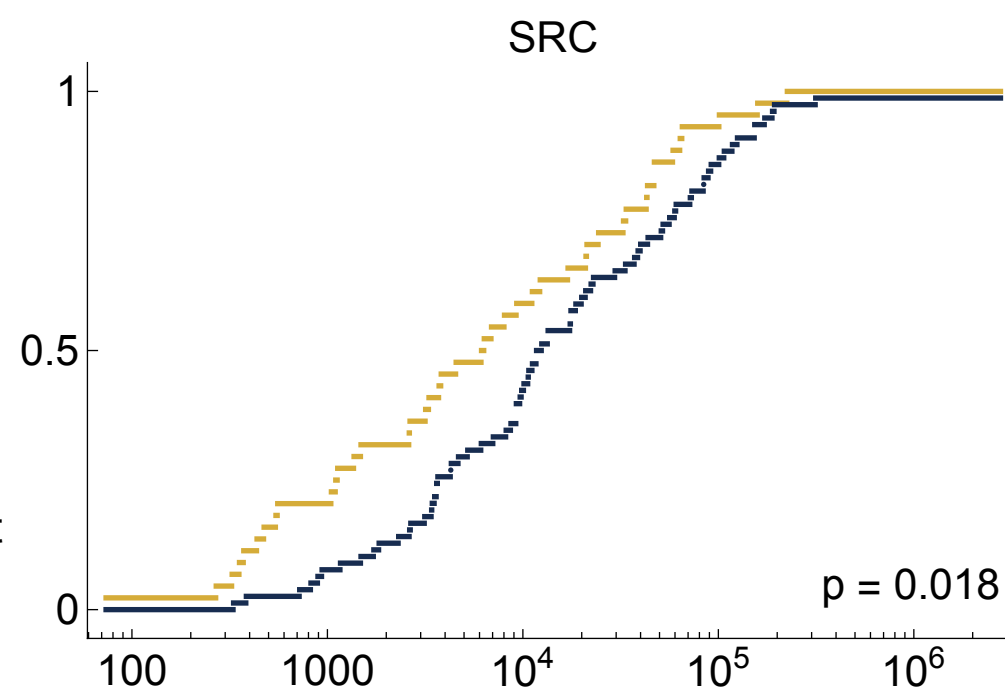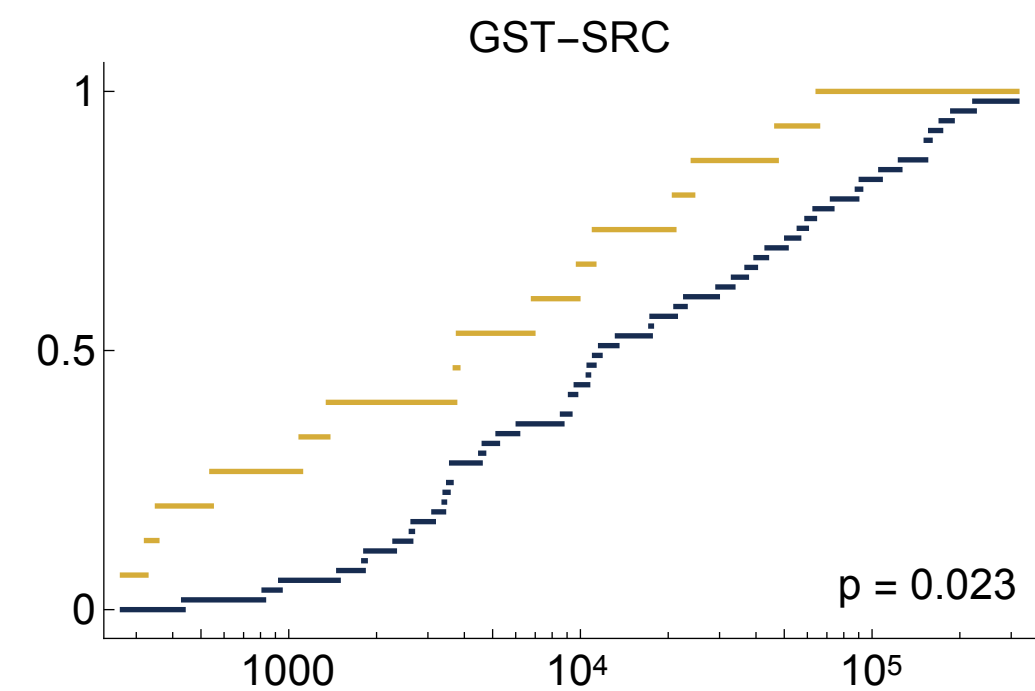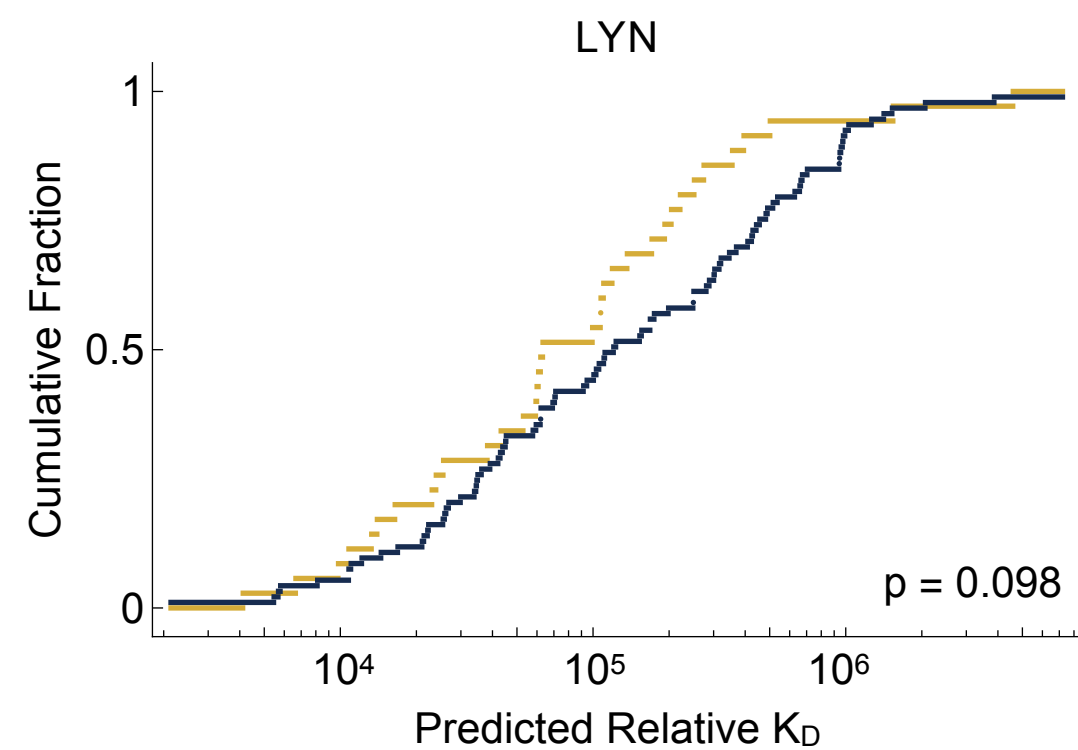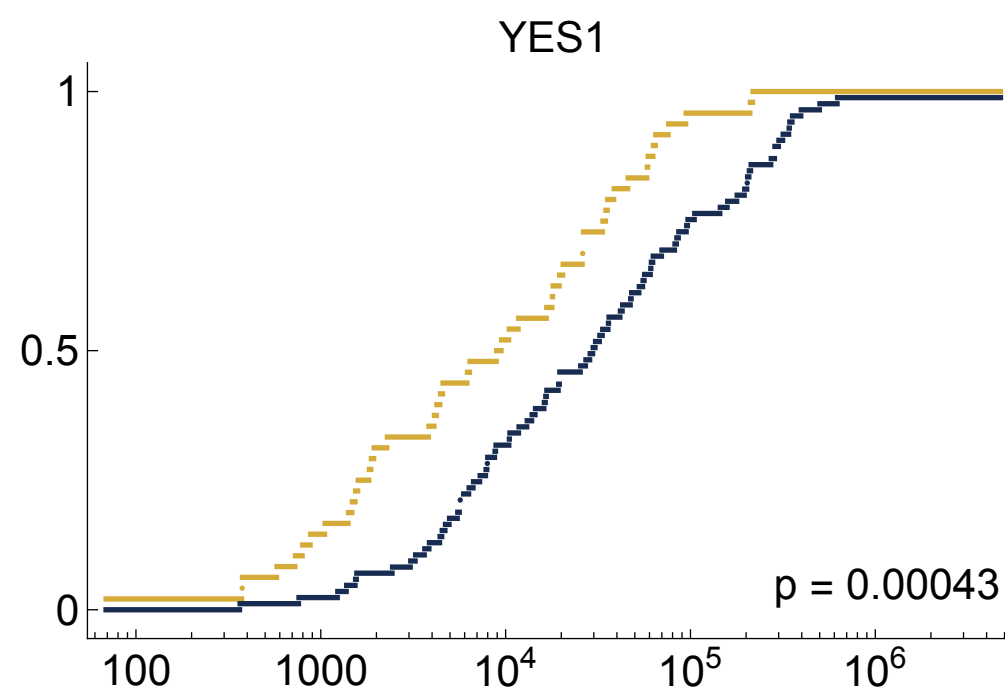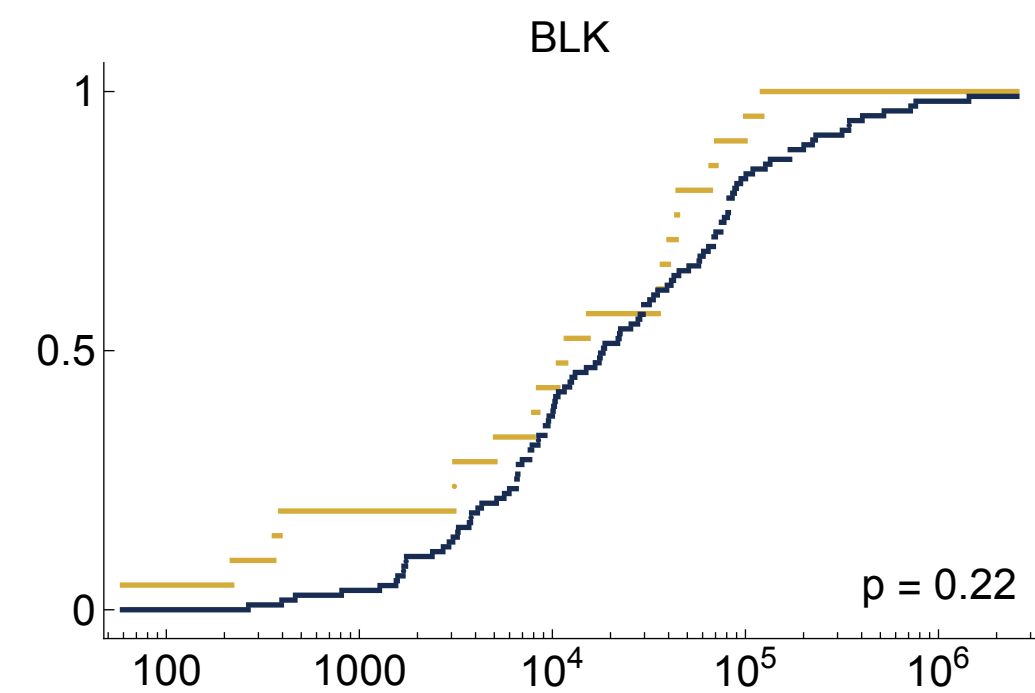

Supplement: Supplementary file 6 — Figure S6: Comparison of model predictions and previous classifications of phosphopeptides as binders and non‐binders. For each SH2 domain, the phosphopeptides classified by (Ronan et al., 2020) were scored using the models in Figures 4a and 5a and the empirical cumulative distribution functions of the resulting relative KD‐values were plotted separately for sequences classified as binders (yellow) and non‐binders (blue). [file PRO-34-e70317-s005.pdf]

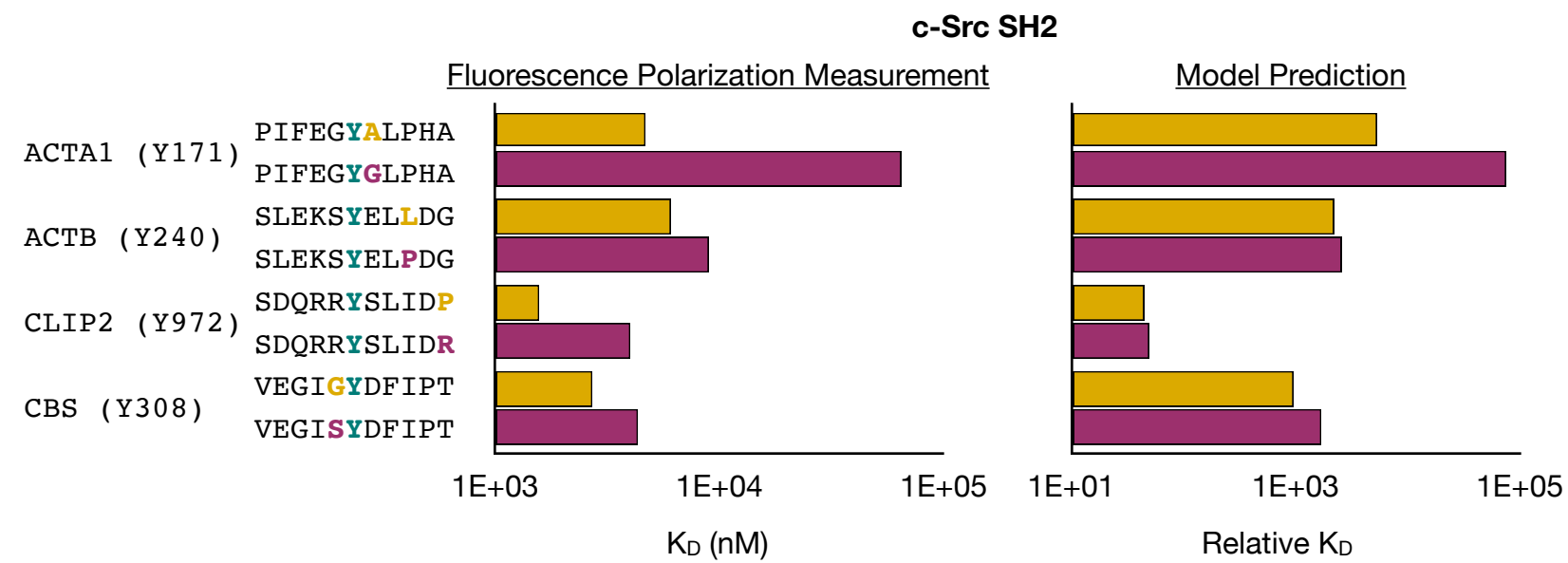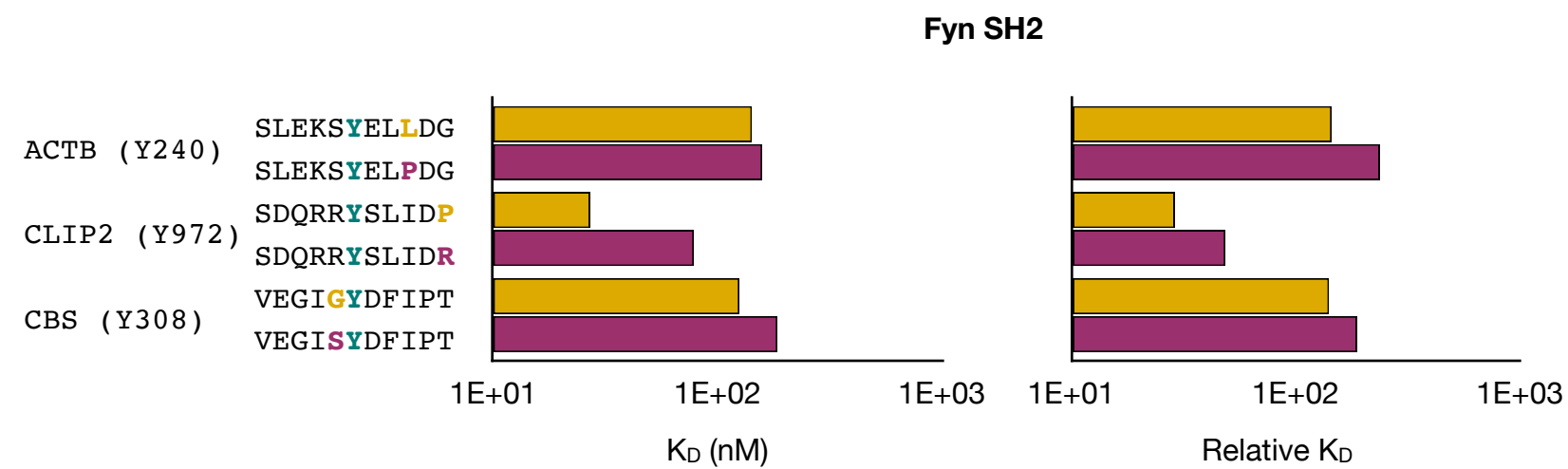

Supplement: Supplementary file 7 — Figure S7: Impact of single‐amino‐acid substitutions on c‐Src and Fyn SH2 binding. The bar charts show the measured KD value (left) and the predicted relative KD (right) for pairs of naturally occurring sequence variants (highlighted letters). The predictions were made using the models shown in Figure 4a. [file PRO-34-e70317-s007.pdf]
